# How social exclusion modulates social information processing: A behavioural dissociation between facial expressions and gaze direction

**DOI:** 10.1371/journal.pone.0195100

**Published:** 2018-04-04

**Authors:** Francesco Bossi, Marcello Gallucci, Paola Ricciardelli

**Affiliations:** 1 Department of Psychology, University of Milan – Bicocca, Milan, Italy; 2 NeuroMI: Milan Center for Neuroscience, Milan, Italy; University of Bologna, ITALY

## Abstract

Social exclusion is a painful experience that is felt as a threat to the human need to belong and can lead to increased aggressive and anti-social behaviours, and results in emotional and cognitive numbness. Excluded individuals also seem to show an automatic tuning to positivity: they tend to increase their selective attention towards social acceptance signals. Despite these effects known in the literature, the consequences of social exclusion on social information processing still need to be explored in depth. The aim of this study was to investigate the effects of social exclusion on processing two features that are strictly bound in the appraisal of the meaning of facial expressions: gaze direction and emotional expression. In two experiments (N = 60, N = 45), participants were asked to identify gaze direction or emotional expressions from facial stimuli, in which both these features were manipulated. They performed these tasks in a four-block crossed design after being socially included or excluded using the Cyberball game. Participants’ empathy and self-reported emotions were recorded using the Empathy Quotient (EQ) and PANAS questionnaires. The Need Threat Scale and three additional questions were also used as manipulation checks in the second experiment. In both experiments, excluded participants showed to be less accurate than included participants in gaze direction discrimination. Modulatory effects of direct gaze (Experiment 1) and sad expression (Experiment 2) on the effects of social exclusion were found on response times (RTs) in the emotion recognition task. Specific differences in the reaction to social exclusion between males and females were also found in Experiment 2: excluded male participants tended to be less accurate and faster than included male participants, while excluded females showed a more accurate and slower performance than included female participants. No influence of social exclusion on PANAS or EQ scores was found. Results are discussed in the context of the importance of identifying gaze direction in appraisal theories.

## Introduction

Everyone has experienced social exclusion in different measures. Being excluded, ostracised, or even simply ignored is part of everybody’s life. Thus, everyone knows how painful, crippling, and confusing this experience can be. This pain arises because being excluded is a threat to our need to belong [1], one of the most fundamental human needs.

In psychological terms, social exclusion is defined as being ignored and excluded by someone [2]. Despite subtle definition differences, the terms “ostracism” and “social exclusion” will be used as synonyms in this study. In addition to our personal experience, we know from many studies that social exclusion has specific impacts on our behaviour, cognitive abilities, emotional feelings, and especially our motivation, defined as fundamental needs (see [3] for a complete review).

To explain in a more specific and accurate way how social exclusion manipulations work, we present Cyberball [4,5]. Cyberball is a manipulation-inducing method of mild social exclusion that is very broadly used since it is fast (lasting approximately 3 min) and easy to administer. It has shown very consistent effects, recently collected in a comprehensive meta-analysis [6]. Cyberball indeed showed its efficacy in many different experimental contexts [7–10]. It is a simple ball-tossing game in which the participant is led to believe that he or she is playing online with other people. The participant can be included, receiving one-third of the passes from the other characters, or excluded, receiving only two passes in the beginning of the game and none thereafter.

The effects of social exclusion on cognitive performance are very well known: ostracism causes a significant decrease in different cognitive skills as a consequence of the experienced threat to individuals’ need to belong [3], also defined as a “defensive state of cognitive deconstruction” [11]. This effect was clearly shown by worse performance on IQ tests [12], time interval estimations, proverbs explanations, simple reaction times tasks [11], and purely cognitive tasks such as the antisaccade task [13]. Ostracism was also proved to impair participants’ cognitive abilities of inhibition, self-regulation [14] and attention control [15].

If the effect of ostracism on cognition is often referred to as “cognitive deconstruction”, its effect on emotional feelings is often defined as “emotional (or affective) numbness”. Despite the fact that on an intuitive ground it is easy to think that the dominant reaction to ostracism could be immediate emotional distress, the most common response is emotional insensitivity [3,16], which often leads to a lack of empathy and prosocial behaviour [17]. In addition to cognitive performance (already described in the previous paragraph), Twenge *et al*. [11] also studied the influence of social exclusion on emotions, by using both explicit measures (self-report questionnaires investigating participants’ mood) and an implicit emotion task (word guessing). Although clear differences in explicit measures were not found, the study showed that excluded participants tended to report fewer emotional words in the implicit emotion task, displaying a deficiency in emotional information processing.

Many studies have investigated the consequences of social exclusion on experienced emotions and feelings, but only a few have examined in depth how emotional and social information is processed by an individual who has experienced ostracism. It is well known that social information is usually processed in a specific way, as shown by the activation of selective brain regions or networks [18,19]. In addition, since ostracism is primarily a social phenomenon, one may reasonably expect that it affects social information processing.

The best-known effect of ostracism on social information processing is the so-called “tuning to positivity” [20,21]. In their 2009 study, DeWall and colleagues [20] revealed that socially excluded participants showed an enhanced selective attention to social signals of acceptance, in particular, smiling faces. This effect was tested on several tasks including visual research and visual cueing, and using both behavioural measures and measures obtained with the eye-tracking technique. In the 2011 study [21], they extended these results by showing that social exclusion increased non-conscious positive affect. This process was revealed to be automatic and unconscious. Therefore, the automatic emotion regulation and enhanced selective attention, both tuned to make positive emotions and acceptance-related signals more accessible, were described as protective mechanisms related to positive mental health.

The processes involved in the “emotional/affective numbness” and “tuning to positivity” effects partially overlap, since both affect the processing of emotional information. For this reason, it is difficult to predict *a priori* whether social information processing could be impaired (according to the former effect) or biased towards re-inclusive signals (according to the latter effect) by social exclusion.

Other studies have explored the importance of re-inclusion after experiencing social exclusion: since ostracism threatens people’s fundamental need to belong [1,3], looking for any possible signal of social inclusion may become a priority for these individuals. Socially excluded people are more accurate in discerning real from fake smiles [22], manifesting higher accuracy in detecting re-inclusive signals. In another experiment, socially excluded participants showed worse performance on a cognitive task, but when they knew they could have a chance of being re-included, they performed even better than included participants [13]. This effect was explained as higher motivation to increase their own inclusionary status by demonstrating their worth on the cognitive task. To close the loop, individuals with a greater need to belong tend to be more attentive to and accurate in decoding social cues [23]

Considering the existing work in the literature, it is clear that the effects of social exclusion on social information processing still need to be explored in depth. We know that social exclusion improves the processing of re-inclusive signals, but many questions are still unanswered. How do ostracised people process socially relevant information? For example, a scared face looking away from us is a socially relevant stimulus that may warn us about an approaching danger, even if it is not related to exclusion or inclusion. Other combinations of facial expression and gaze direction may have different meanings to included and excluded participants. What is the role of important personal differences, such as participants’ empathy and gender, in these processes? The aim of this study is to answer these questions about how social information processing is affected by social exclusion.

The facial features to be explored were determined based on appraisal theories of emotions [24–28]. Appraisal theories of emotion hypothesize that emotions are caused by appraisals (evaluations, interpretations, explanations) of a stimulus/event. Different appraisals may lead to different specific reactions in different people. With regard to evaluation of social stimuli (e.g., facial expressions), appraisal theories suggest that the face expresses cognitive processes involved both in the orienting of attention (primarily gaze direction) and in the evaluation of emotion-eliciting events. This relationship implies an interaction effect between perceived gaze direction and perceived facial expression in inferring emotions from the face. Thus, gaze direction plays a key role in interpreting emotional expressions, since it becomes part of the overall constellation of facially expressive cues that constitute what we perceive as a facial expression [29].

Many studies have evaluated these theories, especially testing their validity regarding the appraisal of facial expressions. These studies revealed the importance of the interaction between facial expression and gaze direction in the interpretation of the emotional value of faces [29–31] and in the updating process in working memory [32,33].

We decided to study the processing of faces with different emotional expressions and gaze directions after social exclusion or inclusion. The processing of these stimuli was tested in two different tasks: emotional expression recognition or gaze direction recognition. By doing so, we aimed to explore if there were any differences in explicitly and implicitly processed features (i.e., emotion and gaze direction, respectively, during emotion recognition, or vice-versa during gaze direction recognition).

Several hypotheses can be made: since it is known that social exclusion can impair cognitive abilities [11] or enhance social information processing [20], we expected that (i) social exclusion could enhance or impair some or all facial features processing. This hypothesis was bidirectional because it was not appropriate to hypothesize in advance whether the results would have shown an impairment reflecting affective numbness [11] or an enhancement reflecting the increased ability of coding emotional expressions [22]. We also expected that (ii) this enhancement/impairment could be moderated by participants’ empathy. In the literature [17], in fact, the effects of social exclusion on prosocial behaviour were shown to be mediated by participants’ empathy. Therefore, it is reasonable to expect that this construct may be a moderator of the effects of social exclusion on social information processing. Finally, we expected that (iii) this enhancement/impairment was dependent on participants’ gender. Differences between males and females in social information processing have been widely reported [34]: gender differences were found in face perception and emotion recognition (*ibidem*), as well as in gaze-related behaviour [35]. Also the effects of social exclusion have been shown to change depending on participants’ gender, as males and females seem to interpret and respond to social ostracism differently [36]. Since no clear and unidirectional prediction could be made from previous literature, we were simply interested in testing the effects of the Cyberball manipulation on recognition of these four emotions combined with different gaze directions.

## Experiment 1

Experiment 1 was conducted as a preliminary experiment. The participants were required to (i) fill in the Empathy Quotient questionnaire [37] to record a validated measure of empathy, (ii) take part in the Cyberball procedure (one half of the participants excluded, one half included), (iii) fill in the Positive And Negative Affect Scale (PANAS) [38] as a self-report measure of positive and negative emotions felt after social exclusion/inclusion, and (iv) take part in a behavioural task in which they were asked to recognize emotional expressions or gaze direction from displayed faces.

### Methods

#### Power analysis

**Fig 1:**
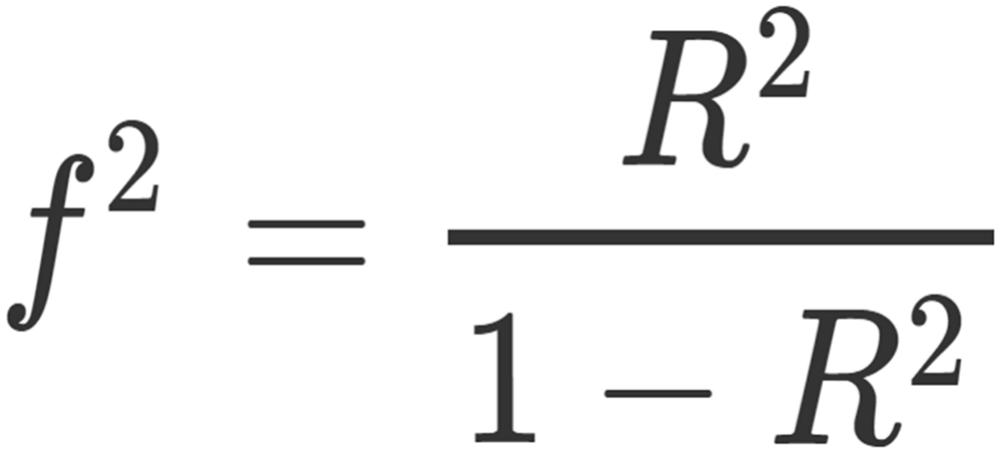
Formula used in order to calculate *f*^2^ effect size from *R*^2^.

First, a power analysis was performed before collecting data in order to determine how many participants were needed to find reliable results. The power analysis was performed for general linear models, considering *f*^2^ as a measure of effect size. *f*^2^ is a measure of effect size based on *R*^2^, as shown in Fig 1, and is considered to be the most reliable effect size measure in Mixed Effects Models [39], which will be used in further analyses. *f*^2^ is typically used as a measure of effect size with continuous dependent variables. In fact, we have also used it (as a function of *R*^2^ changes) for effects computed on participants’ accuracy (binomial) because there is no literature related to other effect size measures in mixed effects models.

To determine the strength of the effect size, we based our decision on a meta-analysis assessing the effects of the Cyberball procedure [5], which found a large effect (d > 1.4) of social exclusion. We decided to consider a medium-large effect of social exclusion in our power analysis, in order to be conservative: *f*^2^ = 0.3 is suggested as a medium-large effect size [40]. Other parameters were set in the analysis: numerator degrees of freedom were set to 1, since we were mainly interested in the main effect of Exclusion and in the interaction effect of Exclusion * Task (both effects have 1 degree of freedom); Alpha (Type I error probability) was set to 0.05; and the power of the test (1 minus Type II error probability) was set to 0.95. As a matter of fact, Exclusion * Task is a between-within subjects effect. We decided to keep *f*^2^ = 0.3 in this effect as well in order to be conservative: if exclusion and task factors are not correlated, the power remains 0.95; if they are correlated, the power increases. This power analysis led to the result of 43.33 degrees of freedom for the denominator, suggesting that testing 44 participants would give a reliable result. We decided to test 60 participants in order to be conservative and to find even more reliable results.

All of the following analyses were performed using (Linear or Generalized Linear) Mixed Effects Models (LMM or GLMM). Since the most appropriate use of these models is under debate (e.g., [41,42]), it is important to specify the decisional pipeline we followed to create the models. First, all of the fixed effects that allowed the model to converge were included. This inclusion typically meant keeping a full factorial model for LMM, but not for GLMM. The same criterion was used to decide whether to include the random effects. In addition, we included only random effects that presented a correlation |*r*| < .80 with other random effects in order to avoid overfitting. Whenever we needed to choose between two random effects that fit the previous criteria but would not allow the model to converge if taken together, we performed two Likelihood Ratio Tests (LRTs) with a null model and chose the model with the best resulting LRT. The significance of each effect was estimated using the Satterthwaite approximation for degrees of freedom in LMMs and performing LRTs with corresponding null models in GLMMs.

#### Participants

A total of 62 participants were recruited mostly among psychology undergraduate students at the University of Milan—Bicocca. Two participants were excluded from the analyses since one could not understand the instructions for the tasks, and one already knew the bogus nature of the Cyberball paradigm (and thus could not be considered a naïve participant). The remaining 60 participants (30 males and 30 females, mean age = 24.9 yr, std dev = 3.79) were included in the analyses. In both Experiment 1 and Experiment 2, participants received course credits for their participation in the study. Both experiments were conducted with the approval from the local Ethical Committee of the University of Milan—Bicocca and in accordance with the ethical standards established in the 1964 Declaration of Helsinki. All participants had normal or corrected-to-normal vision. None of the 60 remaining participants knew about the Cyberball procedure before taking part in the experiment.

#### Materials and procedure

**Fig 2:**
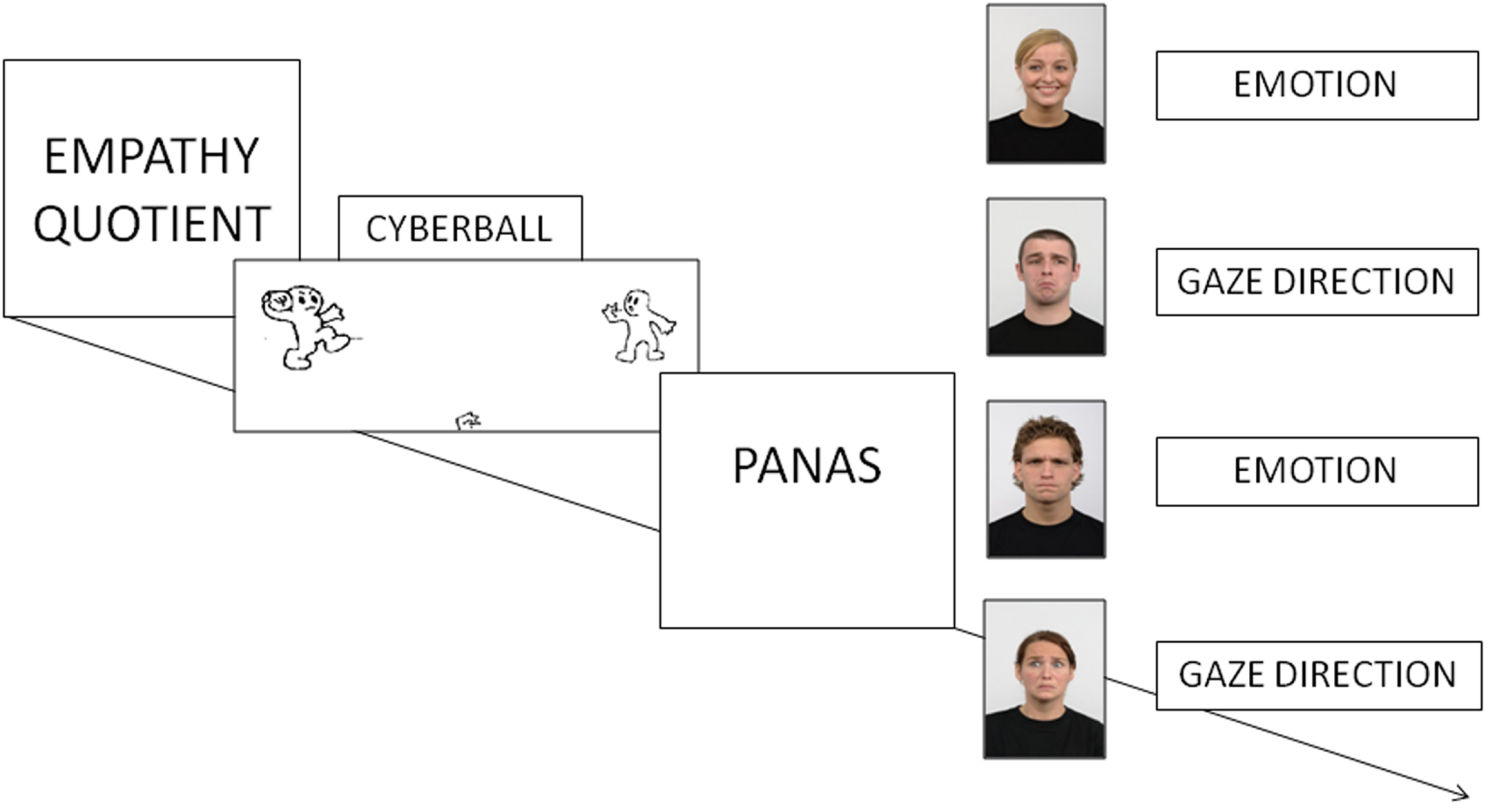
Experimental procedure for Experiment 1. The picture includes four stimuli examples, with different emotional expressions and gaze directions, taken from the Radboud Faces Database (RaFD) [45].

The experiment was carried out in a dimly illuminated room. After reading and signing the informed consent, participants were seated approximately 60 cm away from a 19-inch LCD monitor (resolution: 1024 × 768 pixels; refresh rate: 60 Hz) interfaced with a personal computer with an Intel^®^ Core^™^ i7-3517U 1.90 GHz processor (see Fig 2).

To begin with, participants filled in a computerized version of the Empathy Quotient (EQ) questionnaire [37], presented using E-Prime^®^ 2.0 [43] software. This questionnaire consisted of 60 statements: 40 questions assessing participants’ empathy and 20 filler questions. Participants were asked to respond to each statement choosing among four possible answers (“Strongly agree”, “Slightly agree”, “Slightly disagree”, “Strongly disagree”) using their mouse. The rough scores were recorded.

After completing the EQ questionnaire, participants took part in the Cyberball procedure [5]. The Cyberball game was presented using Inquisit^®^ 4 [44] software. Half of the participants were included during this procedure, receiving one-third of the passes; the other half were excluded, receiving only two passes in the beginning of the game and none thereafter. The number of male and female participants was balanced in each condition (inclusion: 15 males, 15 females, exclusion: 15 males, 15 females).

Then, participants filled in the PANAS questionnaire [38] to assess participants’ felt positive and negative emotions just after the Cyberball game. The questionnaire was presented in computerized form using E-Prime^®^ 2.0 [43] software. A list of 20 adjectives (10 positive and 10 negative, in mixed order) was presented to participants, and they were asked to indicate to what extent they were feeling the emotion described by the adjective at that precise time.

After the PANAS questionnaire, participants began the experimental task. This was performed using E-Prime^®^ 2.0 [43] software. The stimuli used in this task were 96 pictures of Caucasian faces taken from the Radboud Faces Database (RaFD) [45]. The faces were chosen using 8 different identities (4 male actors and 4 female actresses; 4 identities were used only during the training phases): for every identity, 12 pictures were chosen, representing 4 different emotions (happiness, sadness, anger and fear) combined with 3 different gaze directions (direct, left and right). These pictures were presented in a size of 25 x 30 cm (681 x 1024 pixels) and subtended a visual angle of 23° x 26.6°.

The experimental task was divided in 4 alternating blocks in order to avoid learning effects. Depending on the block, participants were asked to perform 2 different tasks: during the first and third blocks, they were asked to identify facial expressions, and during the second and fourth blocks, they were asked to identify gaze direction. Participants could respond by pressing different keys on the keyboard: during the emotion recognition task, they pressed key “Q” for happiness, key “D” for sadness, key “K” for anger and key “P” for fear; during the gaze direction recognition task they pressed key “C” for left, the spacebar for direct and key “M” for right. The keys were marked using adhesive labels representing emoticons (for keys “Q”, “D”, “K”, “P”) or arrows (for keys “C”, “M”, and the spacebar). The association between every key and every emotion/gaze direction was reported in the experimental instructions and by the experimenter him- or herself before the beginning of the experiment. Participants were asked to respond as accurately and quickly as possible. The instructions were shown at the beginning of each block and repeated after every training phase.

Every trial was composed of the presentation of the face (selected in random order) for 1 s, followed by a black screen for maximum of 5 s. Participants could respond during both the presentation of the face and the black screen. If participants had not answered 5 s after the black screen was shown, the trial was considered unanswered, and the following trial was presented. A grey screen lasting 1 s followed every response, before the presentation of the following stimulus.

Every block was composed of 12 training trials and 64 test trials. During the test phase, every stimulus with a direct gaze was presented twice in order to balance the doubled number of stimuli with averted gazes (left and right). After every training phase, participants were given feedback reporting how many trials were answered correctly out of 12. The identity of the stimuli varied across the training phases (one different identity for each training phase), while the 4 identities presented during the test phase were the same across all blocks.

After completing the experimental tasks, participants were debriefed about the experiment (especially about the bogus nature of the Cyberball game) and the experimenter answered their questions.

The entire experiment lasted approximately 25 min. Participants were free to interrupt the experiment in any moment and to take a brief pause between different blocks.

### Results

Statistical analyses were performed using RStudio software (version 1.0.44, [46]), based on R software (version 3.3.2, [47]). Information about specific packages employed in the analyses were reported in Supplementary Materials.

#### Main analyses

The first main analysis for this experiment was a GLMM using the following as independent factors:

- Exclusion (Cyberball manipulation; between subjects; 2 levels: included, excluded),
- Task (experimental task; within subjects; 2 levels: emotion, gaze direction),
- Direction (gaze direction in stimuli; within subjects; 2 levels: direct, averted),
- Emotion (emotion expressed by the stimuli; within subjects; 4 levels: happiness, sadness, anger, fear)

Participants’ Accuracy (binomial: 0–1) was used as the dependent variable. The distribution used in the GLMMs was binomial. Included fixed effects are listed in S1 Table, while included random effects are listed in S2 Table. All tables are available in the supplementary materials. The *R*^2^ calculations of the model were as follows: Marginal *R*^2^ (considering only the variance explained by fixed effects) = 0.24 and Conditional *R*^2^ (considering also the variance explained by random effects) = 0.34 [48].

The statistically significant fixed effect was as follows: Exclusion * Task: χ^2^ (1) = 6.3085, p = .012. All the statistically significant effects not involving any interaction with the factor Exclusion (of primary interest) were reported in Supplementary Materials (for all analyses).

**Fig 3:**
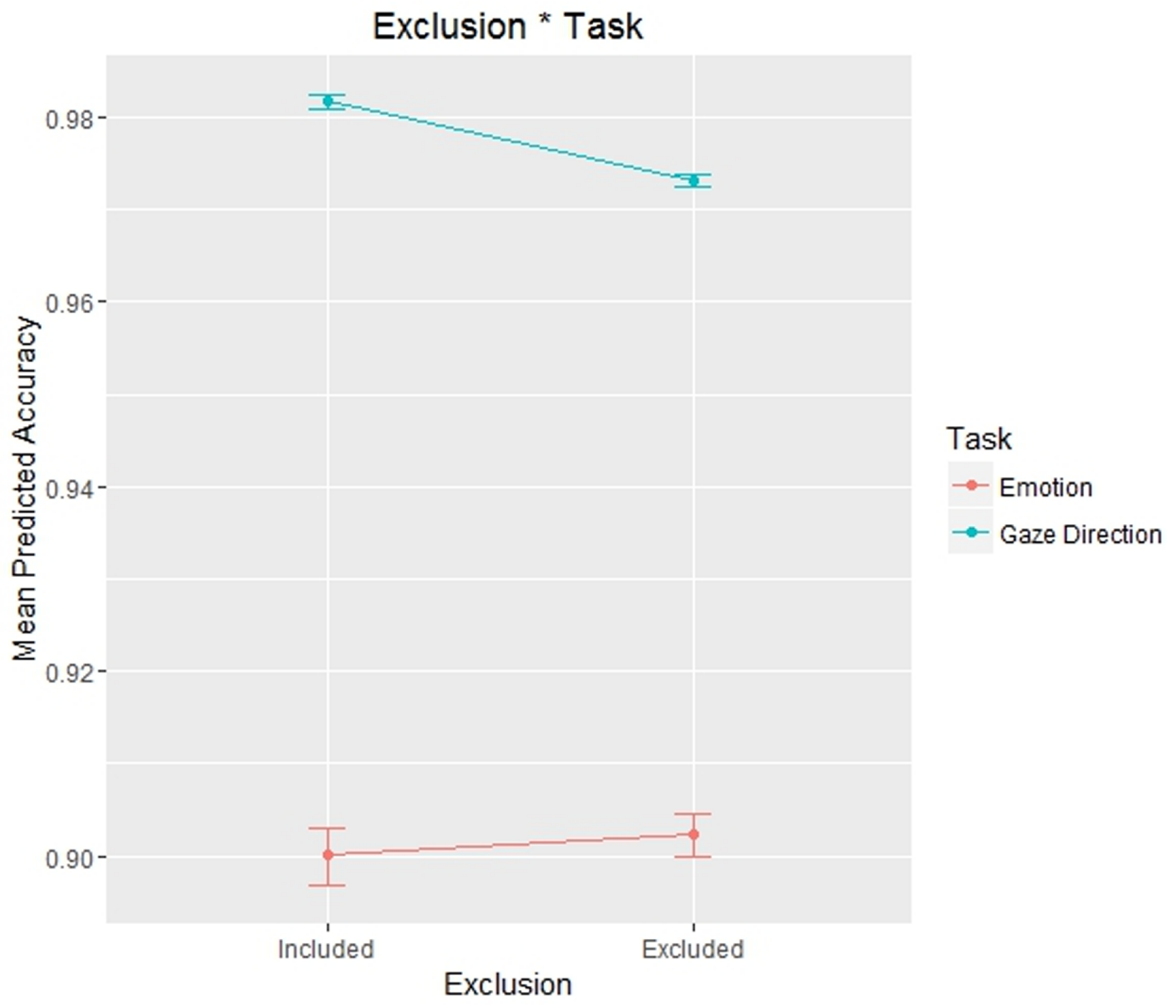
Plot representing the effects of social exclusion in different tasks on participants’ Accuracy in Experiment 1. In this and following plots dots represent the mean value and error bars represent 95% confidence intervals.

The effect Exclusion * Task (Fig 3) is the most interesting one according to our hypotheses, since it is the only statistically significant effect involving an interaction with the manipulation of social exclusion. We then probed this interaction effect with Simple Effect, in order to understand how social exclusion influenced different experimental tasks. A Simple Effect analysis is analogous to post hoc multiple comparisons but is only used to test an interaction effect. In this analysis, the difference between two (or more) groups (in our case, included and excluded participants) is tested under two (or more) conditions (i.e., the two experimental tasks) [49]. In this way, we found a statistically significant difference between included and excluded participants in the gaze direction identification task (included mean = 0.982; excluded mean = 0.973; *z* = 2.227; p = .026), but not in the emotion recognition task (included mean = 0.900; excluded mean = 0.902; *z* = 0.410; p = .682).

The second main analysis for this experiment was a LMM using the same independent variables of the previous model and participants’ response times (RTs; continuous) as the dependent variable. 0.08% of the trials were removed as the RTs exceeded 5 seconds. Trials containing errors (Accuracy = 0) and outliers were removed from this analysis. To identify outliers, we compared the same model performed on 2 different datasets, where trials exceeding the average ± 2 or 3 standard deviations for each participant were removed. The procedure used to choose 2 or 3 std dev as threshold for outliers detection is as following: results of the analyses performed on 2 datasets (in which trials with RTs exceeding 2 or 3 std dev from the mean were removed) were compared. If results did not differ on a significant level we used data included in 3 std dev in order to have more data points to perform a more informative analysis; if results differed in a significant way we used data included in 2 std dev in order to be more conservative. Since the results did not differ on a significant level between the two models, we kept 3 standard deviations as a cut-off. RTs were logarithm-transformed in order to obtain a normal distribution of the residuals. Fixed effects included interactions across all levels among the four independent variables in a full factorial model. Included random effects are listed in S3 Table. The *R*^2^ calculations of the model were as follows: Marginal *R*^2^ = 0.42; Conditional *R*^2^ = 0.58.

The statistically significant fixed effects containing an interaction with the factor Exclusion were as follows: Exclusion * Task: F (1, 13875.2) = 11.2, p < .001; and Exclusion * Task * Direction: F (1, 13885.1) = 6.5, p = .011.

We then probed the Exclusion * Task * Direction effect with Simple Effect, since the 2-way interaction is defined by the 3-way interaction. This analysis showed that in the emotion recognition task excluded participants were slower than included ones, when processing stimuli with direct gaze (included mean = 853 ms; excluded mean = 927 ms; t (61.25) = -2.126; p = .0375). All other comparisons between included and excluded participants across Task and Gaze direction were not significant.

#### Analyses with Empathy Quotient

Then, two different analyses were performed in order to understand the role of the Empathy Quotient score as a moderator of the previous effects, maintaining the previous independent variables. One analysis was performed on participants’ Accuracy (using the structure of the first main analysis) and one on participants’ RTs (using the structure of the second). The first one used the Empathy Quotient score (centred on the overall mean) as a moderator in a GLMM but did not show any significant interaction effects including the EQ score.

The second analysis used the EQ score as moderator and RTs (log-transformed) as the dependent variable in a LMM. Fixed effects included interactions across all levels among the five independent variables in a full factorial model. Included random effects are listed in S4 Table.

The most relevant effect was the significant main effect of the EQ score: t (73) = -2.674, p = .009. This effect showed that participants with higher EQ scores responded more quickly. Another significant effect was the EQ score * Task: F (1, 13848.5) = 26.5, p < .001. We are not going into further detail since this effect did not interact with social exclusion.

#### Analyses with participants’ gender

To understand the role of gender differences as a moderator of the effects of social exclusion, we performed two analyses including participants’ Gender as a moderator: a GLMM on Accuracy and a LMM on RTs.

The first analysis maintained all the previous independent variables and added Gender as moderator. Participants’ Accuracy was the dependent variable. Included fixed effects are listed in S5 Table, while included random effects are listed in S6 Table.

**Fig 4:**
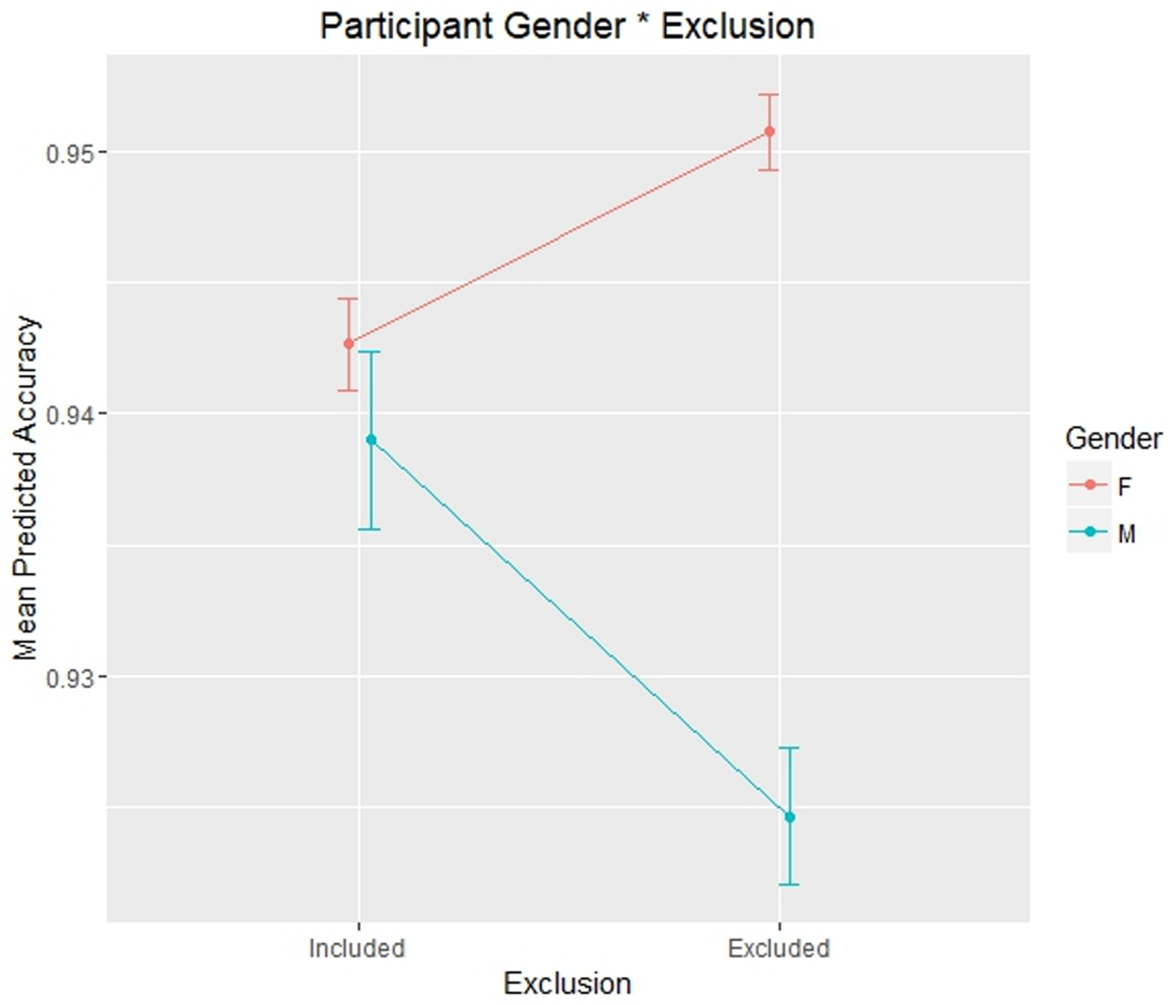
Plot representing the effects of social exclusion in different participants’ gender on participants’ Accuracy in Experiment 1.

The only effect approaching statistical significance and including Gender was as follows: Gender * Exclusion: χ^2^ (1) = 3.591, p = .058 [p-value close to the level of significance]. This effect (Gender * Exclusion; Fig 4) is the most interesting according to our hypotheses and was probed with a Simple Effect: male excluded participants were shown to be significantly less accurate than included male participants (excluded mean = 0.925; included mean = 0.939; z = -2.488; p = .013), while excluded female participants were not significantly more accurate than included female participants (excluded mean = 0.951; included mean = 0.943; z = 0.14; p = .89).

The second analysis was a LMM maintaining the four previous independent variables and adding Gender as a moderator. The dependent variable was log-transformed RTs. In this model, outliers over 3 std dev from the mean were removed, as in the previous models on RTs. Fixed effects included interactions across all levels among the five independent variables in a full factorial model. Included random effects are listed in S7 Table.

The only statistically significant effect containing an interaction with Gender was Gender * Emotion * Exclusion: F (3, 13929.2) = 3.41, p = .017. Post-hoc comparisons were performed on this effect, but no statistically significant comparisons emerged.

#### Analyses on PANAS

Two independent samples t-tests were performed in order to determine if there were any significant differences in PANAS scores between included and excluded participants. In both t-tests, the independent dummy variable was Exclusion, while the dependent variable in one case was Positive Affect score (obtained by summing all positive items in the PANAS), and the dependent variable in the other case was Negative Affect score (all negative items summed). These tests revealed no significant differences in any model examined: Positive Affect: t (58) = -0.459, p = .648; Negative Affect: t (58) = -0.848, p = .4. Additionally, social exclusion appeared to have no influence on participants’ positive or negative affect.

To summarize, in Experiment 1 we found that socially excluded participants were less accurate than included participants in the gaze direction identification task, while excluded participants were slightly slower than included ones during the emotion recognition task, only when processing stimuli with direct gaze. In addition, male excluded participants were shown to be significantly less accurate than included male participants.

## Experiment 2

In Experiment 1, we found that excluded participants were less accurate in gaze direction identification and slightly slower in emotion recognition (while processing stimuli with direct gaze) when compared to included participants. Moreover, excluded male participants were shown to be less accurate than included male participants. All of these effects were considered particularly interesting, taking into account the reciprocal influence of gaze direction in reading emotional expressions and vice-versa [29], and the gender-specific features of social cognition [34], which will be discussed later.

To replicate these effects in a more controlled experimental design, we ran Experiment 2. Specifically, since in Experiment 1 we did not find any effect of exclusion on the PANAS questionnaire (experienced emotions), which in previous studies was often used as a manipulation check for the Cyberball manipulation [11,21,50], we included another measure that has been shown to be strongly associated with social exclusion effects [7,13,51]: the Need Threat Scale [52].

A methodological issue we had to face was that the order of blocks was not varied between participants, despite the fact that we alternated the two experimental tasks in four different blocks to avoid learning effects. This issue could be a potential confound in the interpretation of the results; therefore we decided to present the four experimental blocks in reverse order to half of the participants (balanced for social exclusion and gender) in Experiment 2. Thus, half of them performed the experiment in the order emotion—gaze—emotion—gaze and the other half in the order gaze—emotion—gaze—emotion.

Moreover, we were concerned about the duration of the Cyberball manipulation. If the PANAS and further questionnaires were administered just after the Cyberball game as a manipulation check, we could not be sure of the lasting effects of social exclusion until the end of the experimental tasks. For this reason, we decided to present the questionnaires (Need Threat Scale and PANAS post-manipulation) after the experimental tasks to half of the participants (balanced by exclusion, gender and experimental tasks order). The other half filled in the questionnaires just after the Cyberball manipulation, as in Experiment 1. In this way, we tested for any possible differences in the questionnaires’ scores related to the presentation order (before or after the experimental tasks).

### Methods

#### Participants

In total, 51 participants were recruited based on the criteria described in Experiment 1. None of them participated in the previous experiment. The sample size was smaller than that of Experiment 1, since the power analysis performed for Experiment 1 showed that 44 participants were sufficient to show reliable results, which was confirmed by the effect sizes. Six participants were excluded from the analyses since three could not understand the instructions for the tasks, two already knew about the bogus nature of the Cyberball paradigm (and thus could not be considered naïve participants), and one was an outlier for age (47 years). A total of 45 remaining participants (22 males and 23 females, mean age = 22.9 yrs, std dev = 2.66) were included in the analyses.

#### Materials and procedure

**Fig 5:**
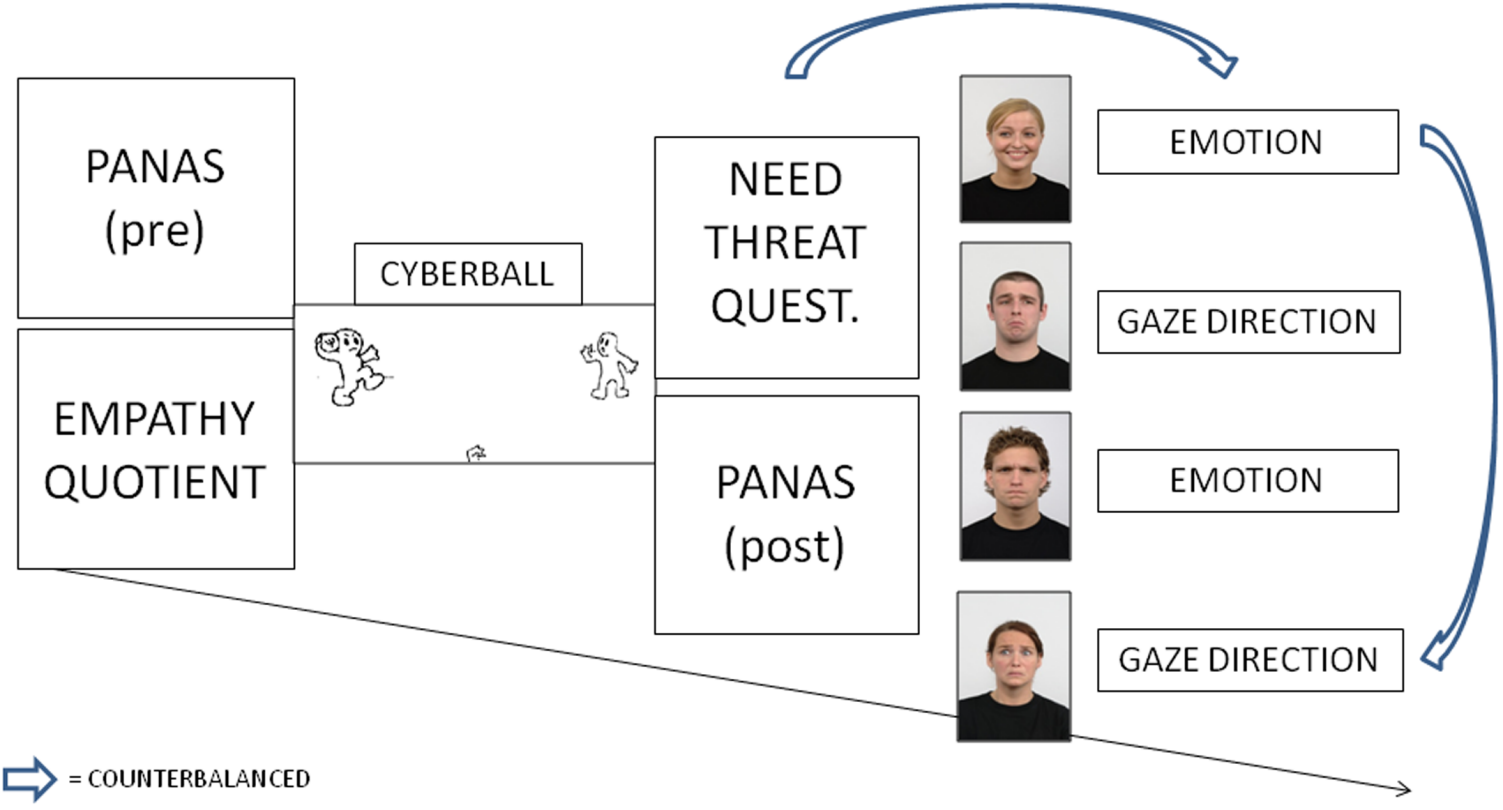
Experimental procedure for Experiment 2. Arrows represent counterbalanced order (across participants) of (i) questionnaires (Need Threat Scale and PANAS post-manipulation) and experimental tasks; and (ii) different experimental tasks. The picture includes four stimuli examples, taken from the Radboud Faces Database (RaFD) [45].

The materials and experimental procedure were the same as in Experiment 1, except the Need Threat Scale (NTS) questionnaire [52] was administered just after the Cyberball manipulation and before the PANAS questionnaire (Fig 5), since the NTS questionnaire is more sensitive than the PANAS and can more accurately discriminate differences between included and excluded participants.

The NTS questionnaire, in fact, is widely used in the literature to measure the effects of social exclusion as a threat to fundamental needs [4,9,51]. The questionnaire was presented in computerised form on E-Prime^®^ 2.0 software [43]. We decided to use it in the version presented by Zadro et al. [10]: 12 statements were presented in random order, 3 for each fundamental need (Belonging, Self-esteem, Meaningful existence, Control). Participants were asked to specify a score from 1 (Absolutely false) to 5 (Absolutely true) for each item. The aggregate score for each fundamental need was then computed by summing the scores of the three related items. Three further questions were included as manipulation checks, as in Zadro et al (*ibidem*): “What percentage of the throws were thrown to you?” (1 = 0%, 5 = 100%), “To what extent were you included by the other participants during the game?” (1 = None, 5 = Completely), “During the Cyberball game I felt…” (1 = Rejected, 5 = Accepted). Furthermore, we also decided to include the PANAS questionnaire before the Empathy Quotient questionnaire. In this way, we had a measure of experienced emotion both before and after the Cyberball manipulation. In this way, we could obtain a more sensitive measure of participants’ emotional experience by computing the difference between scores before and after the ostracism manipulation. Moreover, the NTS questionnaire could not also be used before the manipulation in the same way, since it was specifically concerning the Cyberball game, and thus could not be presented more than once.

The entire experiment lasted approximately 28 min.

### Results

All analyses were performed in the same order and with the same structure (independent variables, moderators, type of models, decisional pipeline for factor inclusion) as in Experiment 1. All differences from previous analyses are noted.

#### Main analyses

The first main analysis was a GLMM on Accuracy. Included fixed effects are listed in S8 Table, while included random effects are listed in S9 Table. The *R*^2^ calculations of the model were as follows: Marginal *R*^2^ = 0.21; Conditional *R*^2^ = 0.27.

**Fig 6:**
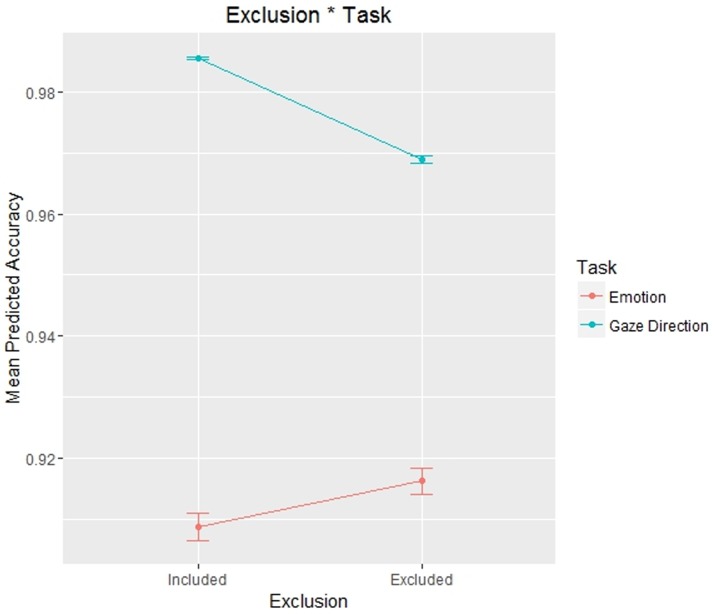
Plot representing the effects of social exclusion in different tasks on participants’ Accuracy in Experiment 2.

The only statistically significant fixed interaction with the social exclusion manipulation was as follows: Exclusion * Task: χ^2^ (2) = 14.638, p < .001 (Fig 6).

As in Experiment 1, we probed this interaction with Simple Effect in order to determine how social exclusion influenced participants’ Accuracy in the different tasks. In the gaze direction identification task, included participants responded more accurately than excluded participants (included mean = 0.986; excluded mean = 0.969; *z* = -2.985; p = .003), while included and excluded participants showed no significant differences in the emotion recognition task (included mean = 0.909; excluded mean = 0.916; *z* = 0.374; p = .709).

The second main analysis was a LMM using participants’ RTs (continuous) as dependent variable. 0.07% of the trials were removed as the RTs exceeded 5 seconds. Trials with Accuracy = 0 and outliers were removed from this analysis. To identify outliers, we performed the same procedure used in the previous experiment. Since the results differed (some effects changed in a significant way) between the models when keeping 2 *vs*. 3 std dev from the mean, we kept 2 standard deviations as a cut-off in order to be more conservative. RTs were logarithm-transformed in order to obtain a normal distribution of the residuals. Fixed effects included interactions across all levels among the four independent variables in a full factorial model. Included random effects are listed in S10 Table. The *R*^2^ calculations of the model were as follows: Marginal *R*^2^ = 0.34; Conditional *R*^2^ = 0.62.

**Fig 7:**
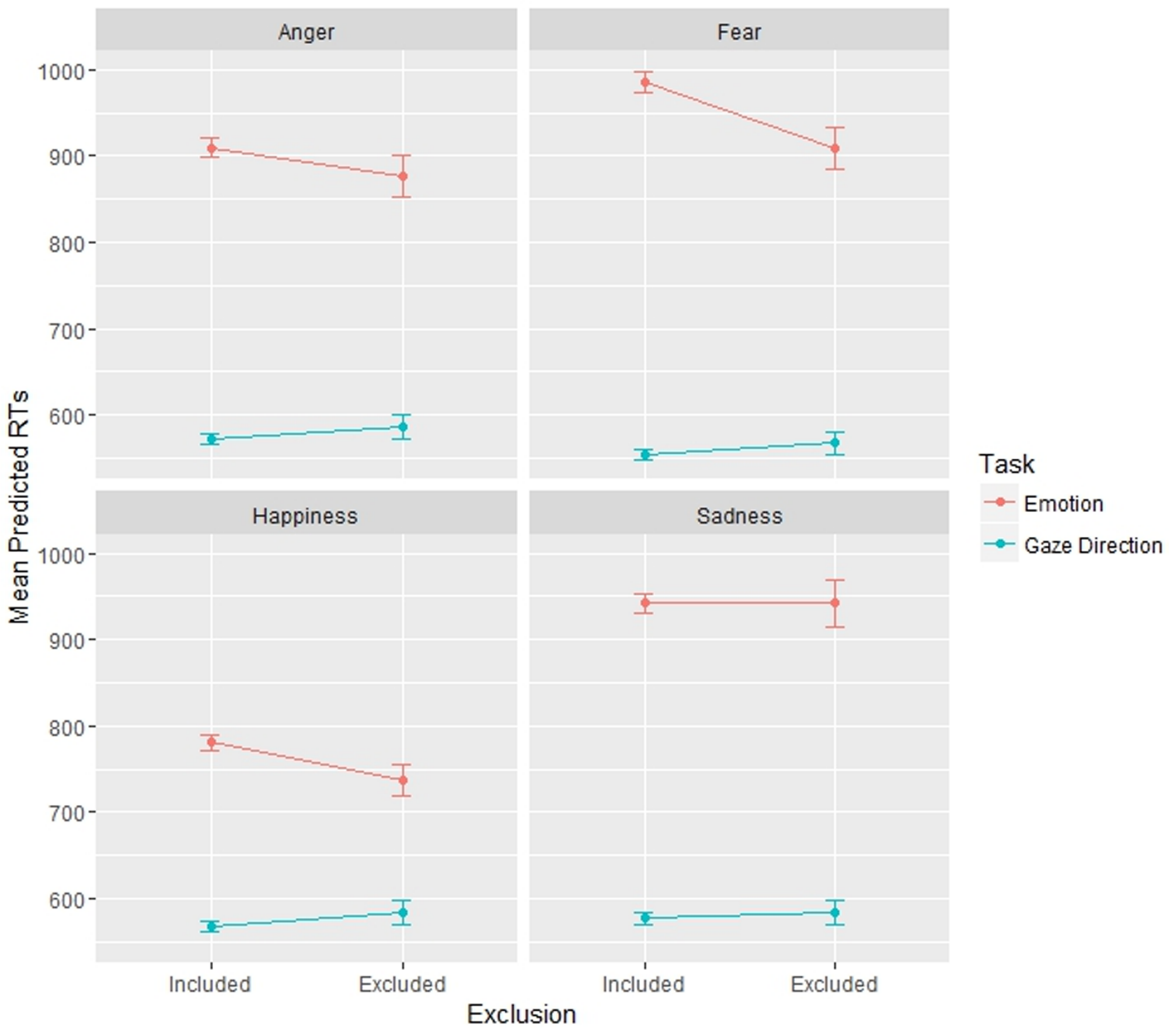
Plot representing the effects of social exclusion in different tasks on participants’ Response Times in Experiment 2, divided by emotions expressed by stimuli.

The statistically significant fixed effects involving Exclusion were as follows: Exclusion * Task: F (1, 10289.9) = 62.9, p < .001; and Exclusion * Task * Emotion: F (3, 10289.6) = 3.8, p = .010 (Fig 7).

We then probed the Exclusion * Task interaction effect with Simple Effect, but the multiple comparisons revealed no significant differences.

A Simple Effect analysis on the 3-way interaction Exclusion * Task * Emotion (Fig 7) revealed very interesting trends. To explore these trends more in depth, four different models were created, one for each emotion. All of these models were analogous to the main one: LMMs, with Intercept | Subject and Direction | Subject as random effects, same independent and dependent variables, except for Emotion, as only one emotion was considered in each model. These models revealed a significant effect of Exclusion * Task for all emotions (Happiness: F (1, 2687.01) = 24.7, p < .001; Anger: F (1, 2454.08) = 13.73, p < .001; Fear: F (1, 2453.59) = 35.83, p < .001), except for Sadness, which did not show this effect as statistically significant (F (1, 2458.36) = 1.91, p = .167).

#### Analyses with Empathy Quotient

Two different analyses, analogous to those carried out in Experiment 1, were performed in order to understand the role of the Empathy Quotient score as a moderator of the previous effects. One analysis was performed on participants’ Accuracy and one on participants’ RTs. The analysis on Accuracy was a GLMM that used the Empathy Quotient score (centred on the overall mean) as moderator. This model showed no significant interaction effects including EQ * Exclusion.

The second analysis considered RTs as dependent variable in two LMMs using the Empathy Quotient score (centred on the overall mean) as moderator. This model did not include any significant interactions including EQ * Exclusion.

#### Analyses with participants’ gender

As in Experiment 1, two analyses were performed in order to understand the role of participants’ Gender as a moderator of the previous effects.

The first analysis is a GLMM using participants’ Accuracy as the dependent variable and Gender as a moderator. Included fixed effects are listed in S11 Table, while included random effects are listed in S12 Table.

**Fig 8:**
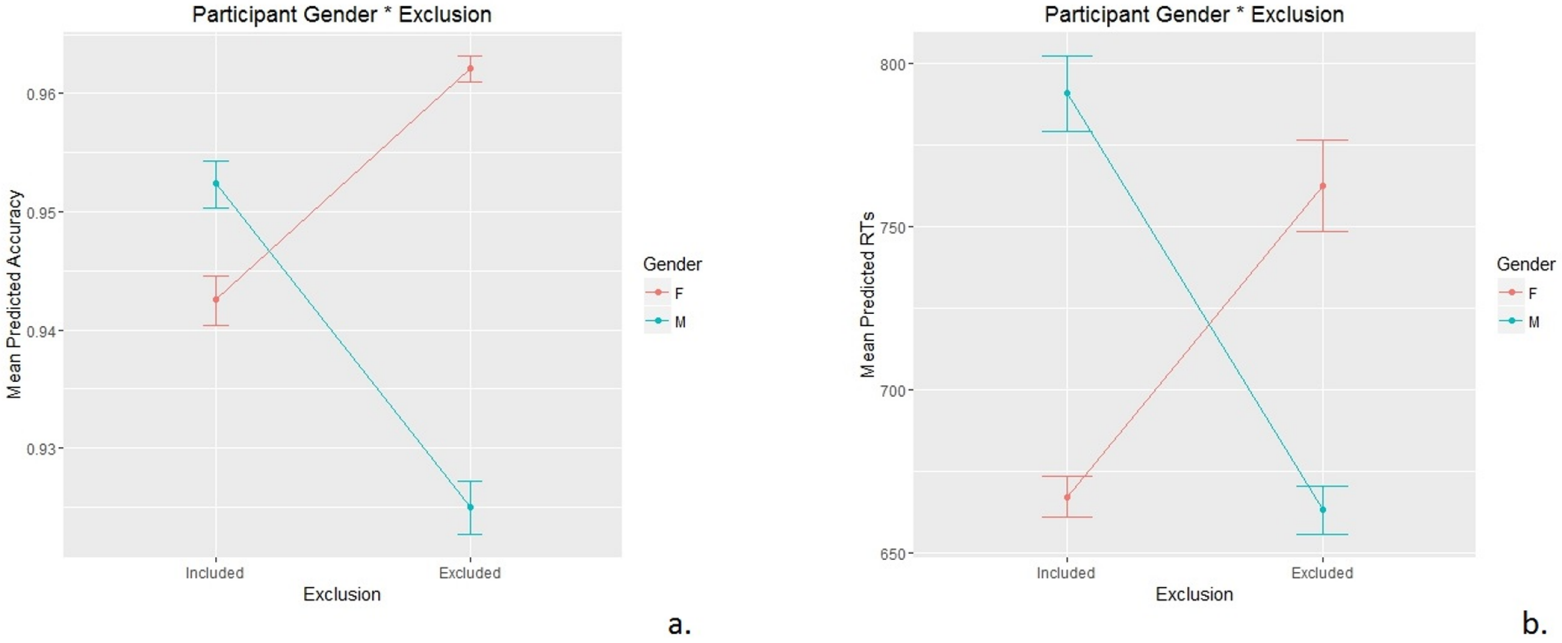
A.: Plot representing the effects of social exclusion in different participants’ gender on participants’ Accuracy in Experiment 2. B.: Plot representing the effects of social exclusion in different participants’ gender on participants’ Response Times in Experiment 2.

The statistically significant fixed effects including Gender were as follows: main effect of Gender: χ^2^ (10) = 22.064, p = 0.015; Gender * Exclusion: χ^2^ (1) = 9.5551, p = .002. The main effect of Gender showed that female participants (mean = 0.951) were generally more accurate than male participants (mean = 0.939). The interaction effect Gender * Exclusion (Fig 8a) was also the most interesting for our hypotheses, since it confirmed the trend we found in Experiment 1. This effect was probed with a Simple Effect: excluded male participants were shown to be significantly less accurate than included male participants (excluded mean = 0.925; included mean = 0.952; z = -3.574; p < .001), while excluded female participants were not significantly more accurate than included female participants (excluded mean = 0.962; included mean = 0.943; z = 0.785; p = .432).

The second analysis performed using Gender as a moderator was a LMM using log-transformed RTs as dependent variable. As in previous analyses, outliers over 2 std dev from the mean were removed. Fixed effects included interactions across all levels among the five independent variables in a full factorial model. Included random effects are listed in S13 Table.

The significant fixed effect involving Gender and Exclusion was as follows: Gender * Exclusion: F (1, 41) = 4.215, p = .046.

This interaction effect, which was interesting as it involved social exclusion effect (Fig 8b), was probed with a Simple Effect and showed that excluded male participants were slightly faster than included male participants, showing a trend towards statistical significance (excluded mean = 663 ms; included mean = 791 ms; t = -1.902; p = .064), while excluded female participants were not significantly slower than included female participants (excluded mean = 763 ms; included mean = 667 ms; t = 0.995; p = .326).

#### Analyses of between-subjects variables

The first analysis of between-subjects variables was performed on PANAS questionnaire scores. For each participant, two scores of PANAS Positive Difference and PANAS Negative Difference were computed, subtracting the scores obtained before the Cyberball game from those obtained after. Then, two independent samples t-tests were performed in order to find any significant differences between included and excluded participants on these two scores. Neither showed any statistically significant differences: PANAS Negative Difference: Excluded mean = 2.14, Included mean = 0.54, t (43) = -1.070, p = .291; and PANAS Positive Difference: Excluded mean = -4.81, Included mean = -3.67, t (43) = 0.711, p = .481. Given these findings, we can conclude that social exclusion did not influence PANAS scores.

The second analysis was aimed to test whether there were any significant differences between included and excluded participants in the Need Threat Scale (NTS) questionnaire. Four different scales (one for each fundamental need) were created for each participant by summing the scores of the related items. Four independent samples t-tests were performed in order to find any significant differences in these scales. All of these tests found significantly lower scores for excluded participants: Belongingness: Excluded mean = 4.38, Included mean = 7.92, t (43) = 6.158, p < .001; Control: Excluded mean = 4.67, Included mean = 7.46, t (43) = 5.488, p < .001; Meaningful Existence: Excluded mean = 6.81, Included mean = 9.71, t (43) = 4.249, p < .001; Self-esteem: Excluded mean = 7.52, Included mean = 9.63, t (43) = 2.844, p = .006.

The third analysis was aimed to study the differences in manipulation checks that were used between included and excluded participants. Three independent samples t-tests were performed on the scores obtained by participants in each of the three questions used as manipulation checks. Excluded participants obtained significantly lower scores in all these tests: Accepted: Excluded mean = 1.48, Included mean = 2.67, t (43) = 4.207, p < .001; Included: Excluded mean = 1.05, Included mean = 2.42, t (43) = 6.035, p < .001; and Percentage of throws received: Excluded mean = 1.52, Included mean = 2.25, t (43) = 5.107, p < .001.

The fourth between-subjects analysis was aimed to check if the Cyberball manipulation effects lasted until the end of the experiment. To do so, we looked for any possible differences in NTS scores and manipulation checks due to the order of questionnaires and experimental task presentation. If no differences in the questionnaires between these two groups were found, then we could assume that the manipulation effects lasted until the end of the experiment. Therefore, seven independent samples t-tests were performed on the four NTS scales and on the three manipulation checks, with order of questionnaires as the grouping factor. None of these tests revealed significant differences in any scores due to the order of questionnaires: Belonging: t (43) = 0.73, p = .469; Control: t (43) = 1.627, p = .111; Meaningful Existence: t (43) = 0.61, p = .545; Self-esteem: t (43) = 1.533, p = .133; Accepted: t (43) = 0.985, p = .330; Included: t (43) = -0.679, p = .501; and Percentage of received throws: t (43) = -1.598, p = .117.

To summarize, in Experiment 2 we replicated the results showing that in the gaze direction identification task, included participants responded more accurately than excluded participants, while included and excluded participants showed no differences in the emotion recognition task. In the analysis on RTs, a significant effect of Exclusion * Task was found for all emotions, except for sadness. No significant interaction effect including the EQ score were found. Moreover, excluded male participants were shown to be less accurate and faster than included male participants.

## General discussion

The aim of this study was to explore how social exclusion manipulation can influence social information processing. To this end, two experiments were carried out, in which participants were asked to identify two of the most relevant and interacting features studied in social cognition [29], i.e., facial expression and gaze direction (which varied across all stimuli). Participants were asked to perform these experimental tasks after being socially included or excluded by means of the Cyberball paradigm [5]. To replicate the results found in Experiment 1 in a more robust experimental design and to be confident about the effectiveness of the Cyberball manipulation, Experiment 2 was designed. This experiment replicated the most important part of the results obtained in Experiment 1, thus corroborating them.

We found a significant Exclusion * Task interaction in both experiments, both on participants’ Accuracy and RTs: results on Accuracy showed that socially excluded participants were less accurate in the gaze direction recognition task, while they did not show any significant differences in the emotion recognition task. By contrast, the results on RTs showed no differences between included and excluded participants in any of the two tasks, thus confirming some influence of social exclusion on social information processing, as hypothesized. Considering that faces can potentially show signals of re-inclusion (or further threat) through a combination of emotional expressions and gaze direction [53], one could have expected higher deployment of attentional resources in processing these cues after social exclusion. In contrast, no enhancement was found, but lower accuracy for excluded participants was detected. This result is consistent with the “deconstructed state” [3] found by Twenge *et al*. [11]. In this study, these authors found effects of cognitive deconstruction (lower performance in cognitive tasks) and emotional numbness (deficiency in emotional information processing) in socially excluded people. According to our results, these phenomena seem to extend to a “social cognition impairment”, specifically affecting the processing of gaze direction, the main feature that communicates where the other person is attending to [54,55] and possible social re-inclusion [53]. This “social cognition impairment” is a new effect and, to the best of our knowledge, it has not been reported before. It shows that excluded people enter into the deconstructed state induced by ostracism, which impairs their ability to deploy their own attentional resources in processing potentially re-inclusive cues. Therefore, the numbness induced by ostracism does not only concern cognitive and emotion-related abilities. Exclusion seems to elicit an effect linked to refusal. That is, ostracized people experience more difficulties in processing signals concerning other people’s attention. This refusal effect could be due to threatening people’s need to belong [1]. In other words, if people were excluded, they did not belong to that group; thus, they did not pay attention and process potentially re-inclusive signals from the faces they saw.

Our results did not show an overall impairment in both tasks, but we did find a specific decline in gaze direction judgement, which suggests a dissociation between the two tasks, implying the involvement of different processes. As stated before, gaze direction plays a key role in interpreting the meaning of expressions, since it becomes part of the configuration of different cues that constitute what is perceived as a facial expression [29]. The gaze is a fundamental feature in the appraisal of emotional expressions because it conveys their meaning [31,32]. Its fundamental role in conveying the meaning of the facial expression to the observer is most likely the reason why gaze direction recognition is specifically impaired by social exclusion (“social cognition impairment”), thus damaging its crucial processing. Some recent work has shed new light on the effects of ostracism on the perception of gaze direction [56], showing that gaze direction processing is altered in socially excluded participants.

These results may seem in contrast with previous studies. DeWall and colleagues [20], for instance, found that ostracized participants increased selective attention towards re-inclusive social signals: they were faster in identifying smiling faces within a “crowd” of discrepant faces, fixated more of their attention on smiling faces and were slower to disengage their attention from smiling faces in a visual cueing experiment. According to these findings, one could hypothesize an enhancement of gaze processing, given the potentially re-inclusive nature of this feature, discussed above. Nevertheless, in our experiment gaze was not always an index of re-inclusion, e.g., angry or fearful faces with direct gaze were a signal of further exclusion, rather than re-inclusion. Since we found no specific effects for smiling faces, we cannot consider our results in contrast with this previous study. Moreover, in the study cited above, social exclusion was elicited by the “Future alone” procedure, which was often found to elicit different (sometimes even opposite) effects from the Cyberball game [57]. Another study by Böckler and colleagues [58] may seem to present results in contrast with our study: using a very clever manipulation, the authors elicited social exclusion by means of a procedure similar to Cyberball, but consisting of gaze exchanges instead of ball tossing passes. In this study, eye-tracking results revealed that excluded participants increased the number and duration of fixations to the player who could potentially reintegrate them. Despite being intriguing, this study does not show an effect of explicit processing of gaze direction, but only an increase of attention directed towards potentially re-inclusive actors. A “social cognition impairment” may also result in longer fixations to faces needed to process gaze direction, because social exclusion had “numbed” participants’ ability to process gaze. In this case, more and longer fixations may also be an index of damaged social processing. Therefore, we cannot conclude that these results are not in line with ours, until a study considering both behavioural and eye-tracking results of gaze direction processing will be performed. Finally, also a study by Wilkowski and colleagues [59] showed results potentially in contrast with our findings: this study revealed that individuals with low sense of belongingness showed increased gaze-triggered orienting, a form of social monitoring. It is important to note, however, that in their study, the authors “*tested whether low belongingness increases […] gaze-triggered orienting*. *Low belongingness was operationalized either in terms of low trait self-esteem (Studies 1a and 1b) or in terms of the priming of rejection-related thoughts (Study2)*”. As the authors pointed out in the introduction, low belongingness is a consequence of long-term chronic social exclusion (also considering the measurement of self-esteem in terms of “trait”), leading to increased monitoring of the social environment. On the other hand, in our study we investigated the influence of acute social exclusion (elicited by the Cyberball game) on participants’ ability to detect social signals. In the literature, it is known that short-term and long-term social exclusion can lead to different (even opposite) effects [52,60] and this can account for the discrepancy between our results and those by Wilkowski and colleagues.

However, this “social cognition impairment” is a novel result. It seems to be robust, given the internal replication across Experiments 1 and 2, resulting in extremely similar effect sizes. Notwithstanding, this novel result needs to be corroborated in future studies.

It is important to acknowledge that this specific effect on gaze direction recognition is small (~ 1–2% on participants’ Accuracy) but extremely consistent. It was coherent between Experiment 1 and 2, both in direction and in effect size. Moreover, the variance in participants’ Accuracy in gaze direction recognition was very low: the variance pooled across experiments was 0.0003 for included participants and 0.0008 for excluded ones. Such a small variance can also explain why mixed effects models with Task as random effect could never converge: if within-task variance is so small, it is almost impossible to capture it with a random effect. Therefore, we can conclude that the social exclusion manipulation generates an apparently small, but very consistent, effect. In addition, gaze-related effects (e.g., gaze cueing) are very small (i.e., differences typically smaller than 100 ms) but extremely constant in direction and size [61,62]. Since gaze direction processing is a low-level ability, participants very often reach a ceiling effect in their Accuracy in gaze-related tasks, giving approximately 100% correct responses (*ibidem*). In these cases, an effect creating a difference of just 1–2% can represent a very effective manipulation.

The lack of statistically significant differences between included and excluded participants in RTs for both tasks is probably due to the large variation of RTs among different participants and different categories of stimuli and to the sensitivity of the measure in these experiments.

Next, it is important to consider the results in light of the appraisal theories [31]. In Experiment 1, the increase in excluded participants’ RTs in emotion recognition was mainly due to stimuli with direct gaze; in Experiment 2, the stimuli expressing all emotions but sadness presented the Exclusion * Task interaction effect. These results showed that modifying these social features (facial expression and gaze direction) modulated the previous effects of social exclusion. The result found in Experiment 1 seems to be counterintuitive given the “Tuning to positivity” effect reported in the literature [21], since direct gaze is a potentially re-inclusive signal, and stimuli with direct gaze are processed slower by excluded participants during emotion recognition. However, this result is much clearer considering the “social cognition impairment” effect caused by social exclusion: on the one hand, faces with direct gaze have a much stronger social meaning to the observer and, thus, are processed in a faster way in the appraisal of faces by included participants. On the other hand, excluded participants appear to be socially numb; therefore, they do not show this advantage for “socially enhanced” stimuli with direct gaze. The second result is even more interesting considering this hypothesis: stimuli conveying sadness do not show an Exclusion * Task interaction effect. While all the other emotions we considered have strong social meanings and are perceived either as a reward (happiness) or a possible threat (anger and fear), the processing of sadness does not bring an evolutionary advantage or have any survival value (i.e., avoiding dangers or obtaining rewards) and, thus, is less socially relevant [63]. Considering the social value of the other emotions, they present the “social cognition impairment”, while sadness is not conveying socially relevant information and thus does not show any specific advantage or disadvantage for excluded participants.

As far as hypothesis (ii) is concerned, that the effects of social exclusion are moderated by participants’ empathy, our results showed no clear effects of moderation by the Empathy Quotient scores in both experiments. In Experiment 1, participants higher in EQ tended to be faster in all tasks. The interpretation of this effect is very intuitive, since empathy by definition is related to social information processing [37]; therefore, people higher in empathy are expected to perform more quickly in tasks involving the processing of social information. It also indicates that EQ is a reliable measure of the construct of empathy. This result, however, was not replicated in the second experiment, probably because of the smaller sample size, noisier data, and a slightly different experimental design. However, the effects of social exclusion did not appear to be moderated by participants’ empathy. Even though, in previous literature, empathy was shown to play an important role in social exclusion [17], this was not true in our study, probably because of the apparently small effect size of the “social cognition impairment”, discussed above. Future research is needed to address specifically the role of empathy in the effect of social exclusion on gaze direction identification.

Regarding our third hypothesis about an effect of gender differences [34] (see [36] for an example of the different effects of ostracism), our results showed that a difference between male and female participants in exclusion effects emerged clearly on both Accuracy and RTs in Experiment 2, while it almost reached statistical significance on Accuracy data in Experiment 1. In other words, excluded male participants overall tended to be less accurate and faster than included male participants, while excluded female participants showed a more accurate and slower performance than included female participants. Therefore, the participant’s gender seems to play an important role in the way people respond to ostracism effects. On the one hand, excluded male participants’ performance was in line with the effects reported in the literature in terms of emotional and cognitive numbness [3,13], here extended to include also the “social cognition impairment”. In particular, they showed difficulties in a social cognition task caused by ostracism, as well as low interest in processing social information (as shown by lower RTs) by deploying less attentional resources. On the other hand, female participants seem to present higher motivation to be re-included. In fact, despite the difficulty they found in performing the tasks (indicated by higher RTs) caused by social exclusion to overcome the “social cognition impairment”, excluded female participants put more effort in performing the tasks and had more motivation, as shown by higher accuracy compared to included female participants. This was also the case in Experiment 1, although this difference did not approach statistical significance, probably due to the methodological differences between the two experiments. Overall, these results extend our knowledge on gender differences in response to social exclusion.

A limitation of this study is the lack of a manipulation check concerning the effects of the Cyberball game before and after the game itself. Our best manipulation check on the Cyberball game was the NTS questionnaire. However, because of its nature, this questionnaire cannot be administered both before and after the manipulation. Future research should address this issue by using an implicit measure that is more sensitive than the PANAS (e.g., skin conductance), the administration of which could be repeated more than once within the same experiment. Despite this limitation, in Experiment 2 we found clear evidence of the efficacy of our Cyberball manipulation both after the Cyberball game and after the experimental tasks.

In conclusion, this study investigated for the first time the effects of social exclusion on two fundamental facial features (emotional expression and gaze direction) taken together. Contrary to what is known in the literature, we found that social information processing was not globally enhanced or impaired, i.e., not all social cues were affected in the same way by social exclusion. Gaze processing was specifically impaired by ostracism, probably because it has a crucial role in communicating important social aspects, i.e., not only social attention but also the comprehension of intentions and mental states. Moreover, this study also showed that male and female participants seem to react differently to social exclusion.

## Supporting information

S1 Table Fixed effects included in the main analysis on Accuracy in Experiment 1. (XLSX)

S2 Table Random effects included in the main analysis on Accuracy in Experiment 1. (XLSX)

S3 Table Random effects included in the main analysis on Response Times in Experiment 1. (XLSX)

S4 Table Random effects included in the analysis using Empathy Quotient scores as moderator on Response Times in Experiment 1. (XLSX)

S5 Table Fixed effects included in the analysis using gender as moderator on Accuracy in Experiment 1. (XLSX)

S6 Table Random effects included in the analysis using gender as moderator on Accuracy in Experiment 1. (XLSX)

S7 Table Random effects included in the analysis using gender as moderator on Response Times in Experiment 1. (XLSX)

S8 Table Fixed effects included in the main analysis on Accuracy in Experiment 2. (XLSX)

S9 Table Random effects included in the main analysis on Accuracy in Experiment 2. (XLSX)

S10 Table Random effects included in the main analysis on Response Times in Experiment 2. (XLSX)

S11 Table Fixed effects included in the analysis using gender as moderator on Accuracy in Experiment 2. (XLSX)

S12 Table Random effects included in the analysis using gender as moderator on Accuracy in Experiment 2. (XLSX)

S13 Table Random effects included in the analysis using gender as moderator on Response Times in Experiment 2. (XLSX)

S1 File Information about packages employed in statistical analyses. (DOCX)
