# Supplementary material for: How social exclusion modulates social information processing: A behavioural dissociation between facial expressions and gaze direction
Source: PLoS One. 2018 Apr 4;13(4):e0195100. doi: 10.1371/journal.pone.0195100 (PMC5884539; doi:10.1371/journal.pone.0195100)
Supplement: S1 File — (DOCX) [file pone.0195100.s014.docx]

**Supplementary Materials**

**Information about packages employed in statistical analyses**

Statistical analyses were performed using RStudio software (version 1.0.44, (RStudio Inc., 2016)), based on R software (version 3.3.2, (R Core Team, 2016)). Information about specific packages employed in the analyses were reported in Supplementary Materials.

Plots were created using the open-source package “ggplot2” (Wickham, 2009). Power analyses were performed using the open-source package “pwr” (Champely, 2016). Mixed Effect Linear Model analyses were run using the open-source packages “lme4” (Bates, Maechler, Bolker, & Walker, 2014) and “lmerTest”(Kuznetsova, Brockhoff, & Christensen, 2015). Post hoc comparisons were performed using the open-source package “lsmeans”(Lenth, 2016). R^2^ computations were performed using the open-source package “MuMIn” (Barton, 2016).

**Statistically significant fixed effects**

**Experiment 1**

**Main analyses**

**Main analysis on Accuracy**

The statistically significant fixed effects were as follows: Emotion: χ^2^ (3) = 195.95, p < .001; Direction: χ^2^ (1) = 39.331, p < .001; Task: χ^2^ (1) = 443.16, p < .001; Emotion * Task: χ^2^ (3) = 72.795, p < .001; Direction * Task: χ^2^ (1) = 5.109, p = .024; and Exclusion * Task: χ^2^ (1) = 6.3085, p = .012.

**Main analysis on Response Times**

The statistically significant fixed effects were as follows: Direction: F (1, 57.9) = 7.4, p = .009; Emotion: F (3, 57.8) = 118.8, p < .001; Task: F (1, 13875.2) = 12691.4, p < .001; Direction * Emotion: F (3, 13858.4) = 3.7, p = .011; Direction * Task: F (1, 13885.1) = 5.5, p = .019; Emotion * Task: F (3, 13894) = 234.5, p < .001; Exclusion * Task: F (1, 13875.2) = 11.2, p < .001; and Exclusion * Task * Direction: F (1, 13885.1) = 6.5, p = .011.

**Analyses with participants’ Gender**

**Analysis using Gender as moderator on Accuracy**

The statistically significant effects including Gender were as follows: main effect of Gender: χ^2^ (11) = 54.494, p < .001; Gender * Task: χ^2^ (1) = 31.944, p < .001; Gender * Exclusion: χ^2^ (1) = 3.591, p = .058 [p-value close to the level of significance].

**Experiment 2**

**Main analyses**

**Main analysis on Accuracy**

The statistically significant fixed effects were as follows: Emotion: χ^2^ (3) = 114.9, p < .001; Direction: χ^2^ (1) = 12.782, p < .001; Task: χ^2^ (1) = 260.53, p < .001; Exclusion * Task: χ^2^ (2) = 14.638, p < .001; Emotion * Task: χ^2^ (3) = 49.099, p < .001.

**Main analysis on Response Times**

The statistically significant fixed effects were as follows: Emotion: F (3, 10289.7) = 117, p < .001; Task: F (1, 10289.9) = 8624.8, p < .001; Direction * Emotion: F (3, 10293) = 5.5, p < .001; Direction * Task: F (1, 10294.3) = 23.1, p < .001; Emotion * Task: F (3, 10289.6) = 140, p < .001; Exclusion * Task: F (1, 10289.9) = 62.9, p <.001; and Exclusion * Task * Emotion: F (3, 10289.6) = 3.8, p = .010.

**Analyses with participants’ Gender**

**Analysis using Gender as moderator on Response Times**

The significant fixed effects involving the Gender were as follows: Gender * Task: F (1, 41) = 5.807, p = .021; Gender * Exclusion: F (1, 41) = 4.215, p = .046.

**References**

Barton, K. (2016). MuMIn: Multi-Model Inference.

Bates, D., Maechler, M., Bolker, B., & Walker, S. (2014). lme4: Linear mixed-effects models using Eigen and S4. *R Package Version*. Retrieved from http://keziamanlove.com/wp-content/uploads/2015/04/StatsInRTutorial.pdf

Champely, S. (2016). pwr: Basic Functions for Power Analysis.

Kuznetsova, A., Brockhoff, P., & Christensen, R. (2015). Package “lmerTest.” *R Package Version*. Retrieved from http://cran.uib.no/web/packages/lmerTest/lmerTest.pdf

Lenth, R. (2016). Least-squares means: the R Package lsmeans. *J Stat Softw*. Retrieved from https://www.jstatsoft.org/article/view/v069i01/v69i01.pdf

R Core Team. (2016). R: A language and environment for statistical computing. Version 3.3.2.

RStudio Inc. (2016). RStudio, integrated development environment for R. Version: 1.0.44. Boston, Massachusetts.

Wickham, H. (2009). ggplot2: elegant graphics for data analysis.
